# Use of a Polyetheretherketone Clasp Retainer for Removable Partial Denture: A Case Report

**DOI:** 10.3390/dj7010004

**Published:** 2019-01-03

**Authors:** Tetsuo Ichikawa, Kosuke Kurahashi, Lipei Liu, Takashi Matsuda, Yuichi Ishida

**Affiliations:** Department of Prosthodontics & Oral rehabilitation, Tokushima University, Graduate School of Biomedical Sciences, 3-18-15 Kuramoto, Tokushima 770-8504, Japan; c301551014@tokushima-u.ac.jp (K.K.); c301751017@tokushima-u.ac.jp (L.L.); matsuda.takashi.1@tokushima-u.ac.jp (T.M.); junchan@tokushima-u.ac.jp (Y.I.)

**Keywords:** polyetheretherketone (PEEK), clasp retainer, removable partial denture, nonmetal clasp

## Abstract

Clasp retainers made of metal alloys may be esthetically unappealing or cause allergic reactions. To investigate alternative materials, we used the nonfiller polyetheretherketone (PEEK) to fabricate the clasp retainer of a removable partial denture for the mandibular bilateral distal free-end abutment of an 84-year-old female. Two years later, few color and texture changes of PEEK were found macroscopically. The rest part and the clasp arm fitted well without any deformation. There were no particular occlusal or periodontal problems. Subjective satisfaction was expressed by both the practitioner and the patient.

## 1. Introduction

The clasp retainer is one of the requisite components of removable partial dentures (RPDs), which are currently used for partial edentulism. The clasp is conventionally made of metal alloys, but most wearers of these dentures show a strong dislike for the metallic color when the clasp is installed in the esthetic zone. In addition, metal allergies present a clinical problem [1,2]. It is therefore desirable to investigate the use of nonmetal materials as substitutes for these clasps.

Materials used for nonmetal denture clasps include polyamide, polyester, polycarbonate, acrylic, and polypropylene, many of which are thermoplastic resins. Physical properties that contribute to the suitability of clasp material include bending strength, flexural modulus, adhesive strength, water absorption properties, abrasion resistance, surface hardness, impact resistance, color stability, compatibility, and ease of processing. However, substitute materials with more esthetic appeal compared with metals frequently do not meet the necessary physical criteria [3,4]. In particular, one problem is material degradation with color and texture changes. Another issue is deterioration in self-cleaning action is another problem for nonmetal denture clasps, which cover the cervical part of the abutment tooth in many cases [5].

Polyetheretherketone (PEEK) is a ketone-based semicrystalline thermoplastic that has been widely used for medical and industrial applications because of its excellent mechanical and chemical resistance properties. In the dental field, its use for crowns, implant superstructures, fixed partial dentures, and RPD frameworks is being investigated [6,7,8,9].

In this case report, we used PEEK to fabricate a clasp retainer for RPDs for the mandibular bilateral distal free-end abutment, although RPD components, such as denture base, retainer, and connector are generally composed of just a thermoplastic resin in the nonmetal clasp denture. After two years of use, we clinically assessed the attributes of this clasp including color and color changes, bacterial adhesion, and compatibility.

## 2. Case Presentation

**Figure 1:**
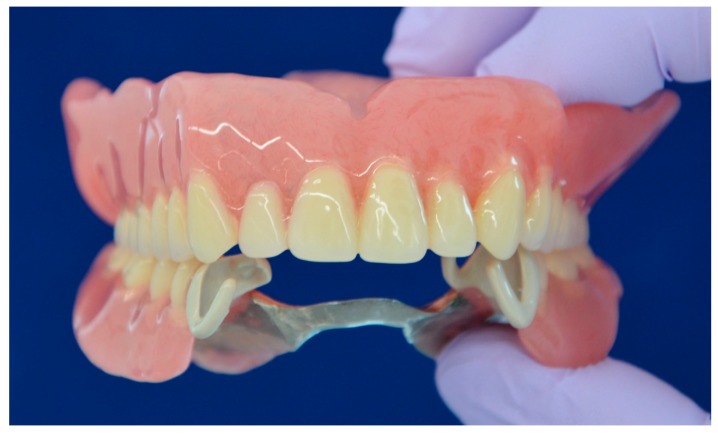
Complete upper denture and removable partial denture with nonfiller polyetheretherketone (PEEK) clasps, at delivery.

**Figure 2:**
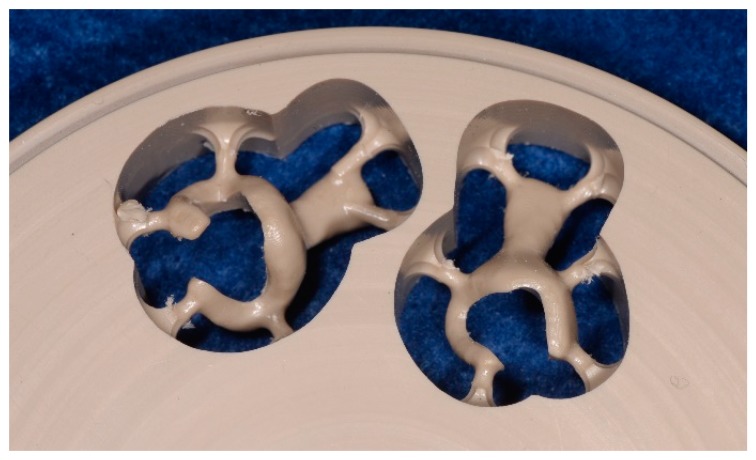
PEEK clasp components milled in the PEEK disk.

An 84-year-old female patient presented to Tokushima University Hospital with only three anterior residual roots in the maxilla and anterior teeth with a bilateral free end saddle in the mandible. Although the crowns in the lower jaw had poor esthetics, the patient was unwilling to receive a revised prosthesis. A treatment plan was devised that involved fitting of a complete overdenture to the maxilla and a RPD to the mandible. Silicone impressions (Exadenture, GC Corporation, Tokyo, Japan) using individual trays and the interocclusal record were obtained according to conventional methods. After trial application of a wax denture, the final denture was made (Figure 1). The clasp retainer was made of nonfiller type PEEK as follows. First, we scanned the working model with a dental scanner; then, we designed the clasp retainer with CAD software (Geomagic Freeform, 3D Systems, South Carolina, USA) and used a milling machine (RXP500 DSC, Roeders BmbH, Soltau, Germany) to shape the clasp from a PEEK disk (JUVORA Dental Discs, Lancashire, UK) (Figure 2). Details of form were modified using technical bars, and polishing was performed with silicone points (Shofu, Kyoto, Japan) and a Robinson bristle brush with polishing paste.

**Figure 3:**
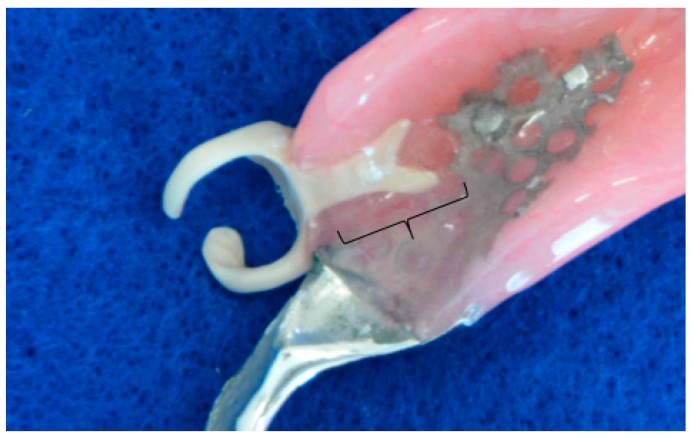
PEEK clasp installed in the denture base. The curly bracket region was treated with sand blasting before flasking.

The adhered surface of PEEK embedded in a resin base was treated using sand blasting with Al_2_O_3_ 50-μm particles (HiBlaster Ovaljet, Shofu, Kyoto, Japan). The denture was then molded using a heat-curing acrylic resin (Acron, GC, Tokyo, Japan) with a conventional flask investment method (Figure 3). Although the clasp apex was primarily positioned in the far zone of the abutment teeth at the fabrication, it was prepared slightly beyond the central line of the abutment at the denture delivery because of the aesthetic problem.

**Figure 4:**
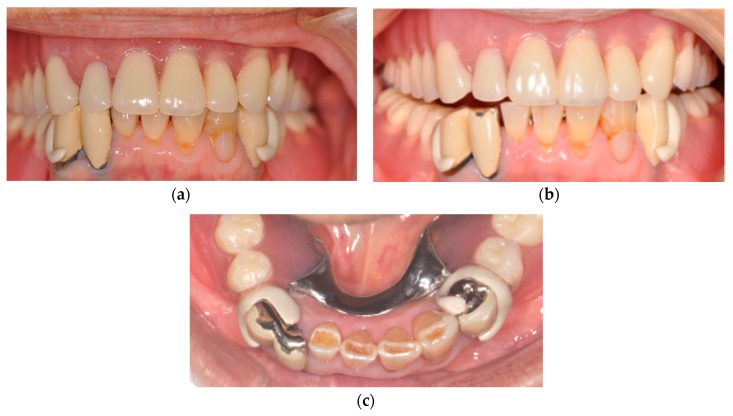
Fit of the PEEK clasps to abutments. (**a**) just after delivery, (**b**) Labial view (two years later), and (**c**): Occlusal view (two years later).

**Figure 5:**
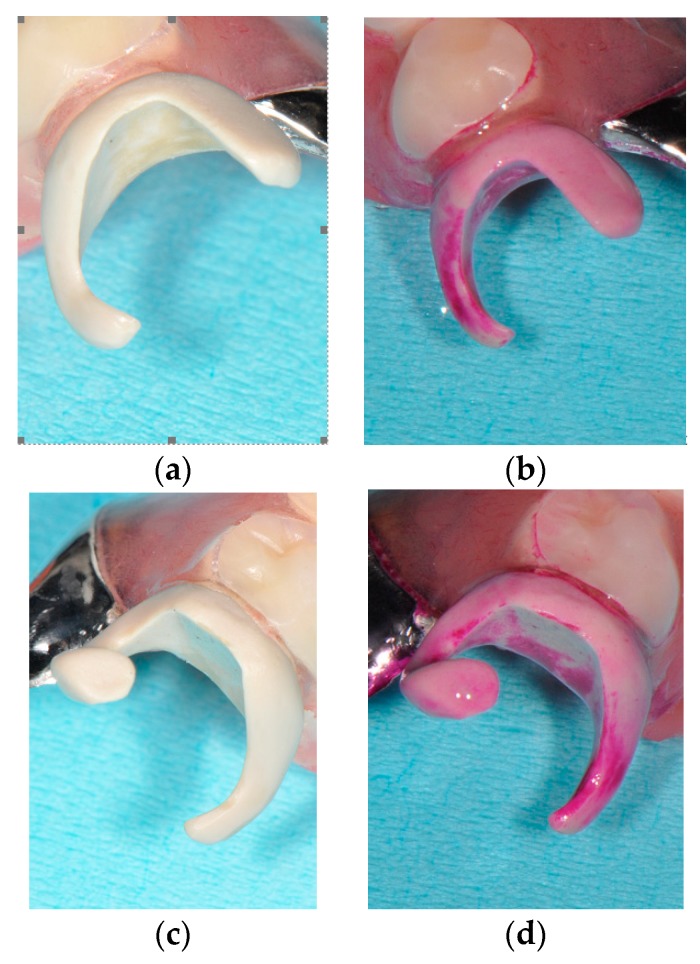
Bacterial adherence on PEEK clasps two years later. (**a**,**c**) before staining and (**b**,**d**) after staining.

**Figure 6:**
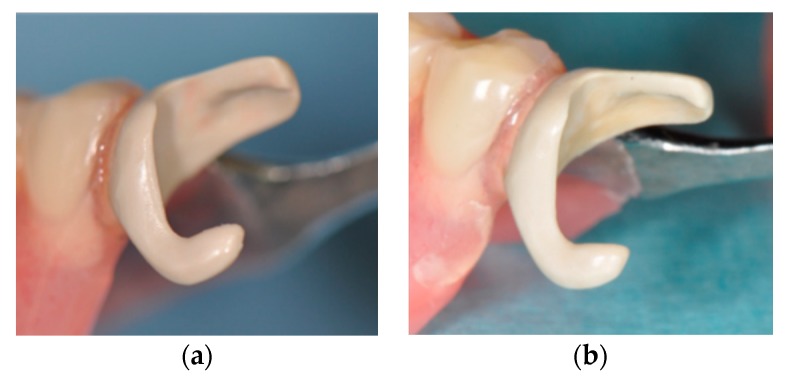
Surface change on the PEEK clasp. (**a**) just after delivery and (**b**) two years later.

Figure 4, Figure 5 and Figure 6 show the PEEK clasps two years after delivery. The patient reported that denture had been rinsed under running water after every meal and immersed into a denture cleanser for the night at bedtime. Few color and texture changes were found macroscopically, and denture plaque adherence around the clasp appeared minimal. However, staining with a plaque-disclosing agent revealed clear denture plaque on both the inside and outside of the clasps. 

The rest part and the clasp arm still fitted well without any deformation, and the participant reported no particular problem with occlusal contact.

No specific mobility of the abutment teeth, and no specific inflammation of the gingiva around the abutment teeth were found. The subjective operational opinion of the practitioner about wearing and detaching of the denture was also reported as good.

## 3. Discussion

This case report on the PEEK clasp involved a follow-up of short duration (two years), but both patient and practitioner were almost satisfied with the outcome. Few color and texture changes were observed, reconfirming the chemical stability and biocompatibility of PEEK, although it has been reported that such changes in other nonmetal clasp materials are occasionally found several months after delivery.

Biofilm formation on the surface of PEEK has been reported to be equal to or lower than that on the surface of conventionally applied abutment materials, such as zirconia and titanium [10]. However, bacterial adherence is easily overlooked, because the colors of denture plaque and PEEK material are similar. Clinical examination revealed no periodontitis or gingivitis in relation to the abutment teeth. The clasp design of PEEK may be similar to that of nonmetal denture clasps considering the mechanical property of PEEK, while the PEEK clasp permits reduction of the coverage/proximity of the cervical part of abutment teeth. Therefore, the gingiva around the PEEK clasp should be monitored with regular check-ups.

Clinical complications around the interface between the metal frame and denture base resin, such as fracture of the denture base and invasion of foreign objects into the gap, often occur in conventional RPDs [11]. In our case study, such complications were not found after two years. The difference in elastic modulus between PEEK and acrylic resin is smaller than that between metal and resin, reducing the likelihood of these interface problems, although there is no chemical adhesion between PEEK and acrylic resin [12].

Esthetically, the color of conventional nonmetal clasp materials is viewed as that of gingiva, while the color of PEEK is that of teeth. The slightly grayish, nontransparent white color of PEEK was more acceptable than the silver color of a metal clasp. Nonetheless, it did not match the tooth or crown color sufficiently. If the PEEK clasp is placed in the esthetic zone, the clasp apex had not better located in the far zone of the abutment teeth, but rather positioned slightly beyond the central line of the crown as we did in our case. Future development of PEEK that matches the tooth color more closely and is slightly more transparent will improve its esthetics.

The elastic coefficient of PEEK is approximately 4 GPa [13], which is considerably less than that of gold alloy (100 GPa) and Co–Cr alloy (200 GPa) [14]. Appropriate retention force and bracing will need to be attained by increasing the thickness and width of the clasp arms in PEEK relative to those of conventional metal clasp. Clasp retentive force is directly proportional to these attributes, and also to elastic modulus and the degree of undercut, and is inversely proportional to the length of the clasp arm [15,16]. Therefore, the shortened and wide clasp arm, with the clasp apex slightly overlapping the center, contributed to improved aesthetics and to increased retentive force. In contrast, the lower flexural modulus than that of metal may be beneficial, because it reduces the risk of excessive force on the abutment tooth [17]. To date, it has not been reported that the PEEK clasp occlusal force distribution was unsatisfactory in this case. Clasp design without coverage of the cervical area, in contrast with conventional thermoplastic resins, is advantageous for periodontal disease and root caries. 

A PEEK clasp has the same disadvantages as other nonmetal clasp materials: difficulty of polishing and adjusting retention capacity. The appropriate polishing procedure of PEEK is under consideration; it is expected to have the same smoothness as that of acrylic resin. It is impossible to adjust the retention capacity due to clasp bending at delivery; therefore, preoperative designing is necessary for a PEEK clasp. 

In conclusion, we have presented a satisfactory outcome of an RPD case utilizing a PEEK clasp retainer over a short observation period. Standardizations of clasp design and laboratory and clinical work will be required in the near future.
